# Influenza vaccine effectiveness among adults aged ≥60 years in northeastern Zhejiang Province, China, 2021–2024

**DOI:** 10.3389/fpubh.2025.1730158

**Published:** 2026-01-16

**Authors:** Zhao Yu, Xinyu Liu, Jiayun Fu, Xiaokun Yang, Yang Liu, Yanru Chu, Jialie Jin, Zenghao Xu, Yanli Cao, Jinren Pan, Shelan Liu, Xiaofei Fu, Tianfeng He, Hangjie Zhang

**Affiliations:** 1 Department of Prevention and Control of Infectious Disease, Zhejiang Provincial Center for Disease Control and Prevention, Hangzhou, Zhejiang, China; 2 School of Public Health, Hangzhou Medical College, Hangzhou, Zhejiang, China; 3 Department of Public Health, Central Hospital of Huashui Town, Jinhua City, Zhejiang, China; 4 Division of Infectious Disease, Key Laboratory of Surveillance and Early-Warning on Infectious Disease, Chinese Center for Disease Control and Prevention, Beijing, China; 5 Department of Infectious Diseases, Jiaxing Center for Disease Control and Prevention, Jiaxing, Zhejiang, China; 6 Department of Infectious Diseases, Ningbo Center for Disease Control and Prevention, Ningbo, Zhejiang, China

**Keywords:** older adults, influenza vaccine, test-negative design, vaccination, vaccine effectiveness

## Abstract

**Background:**

Influenza poses a particularly severe threat to older adults, yet vaccination coverage among this vulnerable population remains suboptimal in China. To address this public health challenge, Zhejiang Province initiated a free influenza vaccination program for older residents starting in 2020. This study evaluated the effectiveness of influenza vaccination in reducing outpatient visits among adults aged ≥60 years during three consecutive influenza seasons (2021–2024).

**Methods:**

We employed a test-negative design (TND) among adults aged ≥60 years presenting with influenza-like illness (ILI) at sentinel surveillance hospitals in two cities in Zhejiang Province from October 2021 to April 2024. Standardized questionnaires were administered to collect demographic and clinical data. Respiratory specimens were tested for influenza virus types and subtypes using RT-PCR. Multivariable logistic regression models were employed to assess factors associated with vaccination status and influenza virus detection, with subsequent estimation of influenza vaccine effectiveness (VE).

**Results:**

A total of 3,796 ILI cases were enrolled, with 644 testing positive for influenza, yielding a positivity rate of 16.97%. The results of multivariable logistic regression analysis showed that age, whether vaccinated in the current year, and whether vaccinated in the previous year were the influencing factors for influenza-positive ILI cases (*p* < 0.05). The influenza vaccination coverage in the current season was 33.14%. The overall VE was 47.21% (95% CI: 35.38 to 56.88%). Subtype-specific VE was 55.81% (95% CI: 34.83 to 70.03%) for H1N1, 40.72% (95% CI: 23.30 to 54.18%) for H3N2, and 55.16% (95% CI: 21.77 to 74.30%) for B/Victoria. Age-stratified VE analysis showed effectiveness of 70.34% (95% CI: 41.47 to 84.98%) among those aged 60–69 years, 49.48% (95% CI: 34.41 to 61.09%) in the 70–79 age group, and 38.34% (95% CI: 10.35 to 57.60%) among individuals aged 80 years and older.

**Conclusion:**

Influenza vaccination provides moderate protection for adults aged ≥60 years, with effectiveness varying by subtype, age, and season, particularly limited in the older population aged ≥80 years.

## 1 Introduction

Influenza is an acute respiratory infection caused by influenza viruses, capable of causing annual epidemics and remains a persistent public health challenge (1). Characterized by high mutability and rapid transmission, influenza viruses pose elevated risks of severe illness and complications among immunocompromised populations, particularly the older adults.

China’s Technical Guidelines for Influenza Vaccination (2023–2024) recommend prioritizing vaccination for high-risk populations (e.g., individuals with chronic diseases or aged ≥60 years) (2). However, as influenza vaccines are not included in China’s national immunization program and require self-payment, coverage rates have historically been low. An earlier study reported that only 3.8% of adults ≥60 years received influenza vaccination, far below the threshold needed for herd immunity (3). In contrast, many developed nations (e.g., Sweden, the UK) have implemented free vaccination policies for high-risk groups or entire populations to improve coverage (4). In China, such policies are currently limited to pilot programs in economically advanced regions like Beijing (5) and Shenzhen (6).

Since 2020, Zhejiang Province has progressively expanded free vaccination eligibility: initially covering adults ≥70 years with local household registration under government livelihood projects, then extending to ≥65 years (2021), and finally to ≥60 years (2024) (7). This policy initiative has been associated with a substantial increase in vaccination coverage at the local level. For example, in Ningbo city, vaccination coverage among older adults increased from 1.14% in the 2017–2018 season to 33.41% in 2022–2023, with coverage specifically reaching 50.03% among the target population aged ≥70 years (8). A district-level analysis in Yinzhou further documented a 29.1% absolute increase in vaccination rates following policy implementation (9).

While previous studies have estimated influenza vaccine effectiveness (VE) among older adults in Zhejiang Province, most were limited to single seasons or individual cities. Multi-season, subtype-stratified, and age-stratified VE data under the free vaccination policy remain scarce. To address this evidence gap, this study aims to estimate the protective effectiveness of influenza vaccination against outpatient influenza infections among individuals aged ≥60 years over three consecutive influenza seasons (2021–2024), utilizing surveillance data from sentinel hospitals in Ningbo and Jiaxing cities of Zhejiang Province. The findings are expected to provide evidence-based insights for optimizing influenza vaccination strategies for the aging population in China.

## 2 Methods

### 2.1 Study population

This study employed a test-negative design (TND), a widely used observational study design for evaluating influenza VE. Influenza-like illness (ILI) patients aged 60 and above who were eligible for the local free influenza vaccination policy and were treated in the influenza surveillance sentinel hospitals in Jiaxing and Ningbo, Zhejiang Province from October 2021 to April 2024 were selected. ILI refers to patients with an acute onset (duration of illness ≤ 7 days), a maximum body temperature of ≥ 37.5 °C, and accompanied by either cough or sore throat. Cases were excluded if they met any of the following conditions: (a) Contraindications to influenza vaccination; (b) Residency in nursing homes or living outside the surveillance area; (c) 7 days between symptom onset and medical visit, or prior use of antiviral medications; (d) inability to communicate effectively with the patient or guardian; (e) Declined to participate or unable to provide informed consent.

### 2.2 Data collection

At the time of patient enrollment, uniformly trained medical staff administered an electronic questionnaire for ILI immediately following specimen collection and prior to any diagnostic testing. The questionnaire collected demographic data, vaccination history, clinical symptoms, specimen details, and laboratory results.

The questionnaire was developed by the research team in collaboration with influenza vaccine experts and the Zhejiang Provincial Center for Disease Control and Prevention (CDC). To ensure validity, a pilot test was conducted with 10 older adults (not included in the final analysis). All data were anonymized, and written/verbal informed consent was obtained prior to participation.

Vaccination records for the study period were primarily retrieved from the Zhejiang Provincial Immunization Registry, a mandatory reporting system that captures influenza vaccinations administered at all accredited healthcare facilities across the province. This registry uses unique citizen identification numbers to ensure accurate individual-level linkage, providing a reliable and nearly complete source of vaccination data. Study participants were classified as vaccinated if the registry indicated receipt of a seasonal influenza vaccine at least 14 days prior to their ILI symptom onset. For the primary analysis, “vaccinated” status was assigned solely based on receipt of the current season’s vaccine.

### 2.3 Laboratory confirmation

Pharyngeal swab specimens were collected from participants and tested for influenza virus nucleic acids using real-time reverse transcription polymerase chain reaction (RT-PCR). First nucleic acids were tested for influenza viruses A (A) and B (B); A-positive specimens then went on to be tested for A (H1N1) and A (H3N2), and B-positive specimens were tested for the B/Victoria lineage and the B/Yamagata lineage, and the cases that were positive for influenza viruses were formed into a case group, and those that were negative for influenza viruses were formed into a control group. Throughout the study period, the trivalent influenza vaccine administered in Zhejiang Province followed WHO recommendations for the Northern Hemisphere. National surveillance indicated that influenza B/Victoria predominated in 2021–2022, A/H3N2 and A/H1N1 co-circulated in 2022–2023, and A/H3N2 was the dominant subtype in 2023–2024 (10, 11).

### 2.4 Statistical analysis

Variable selection for the multivariable logistic regression model was based on biological plausibility, prior knowledge, and associations (*p* < 0.10) observed in bivariate analyses. A TND was employed to estimate influenza VE by comparing influenza positivity rates between vaccinated and unvaccinated individuals. Categorical variables were analyzed using chi-square tests. Logistic regression models were used to identify factors associated with both vaccination status and influenza positivity. Adjusted odds ratio (aOR) with 95% confidence intervals (CI) were calculated after controlling for potential confounders in the final model, which included sex, age group, underlying medical conditions, influenza season, and vaccination status in the previous season. Model fit was assessed using the Hosmer-Lemeshow goodness-of-fit test, which indicated adequate fit (*p* > 0.05). Multicollinearity was evaluated using variance inflation factors (all < 2.0). VE was calculated as 100% × (1 − aOR), with *p* < 0.05 indicating a statistically significant difference.

### 2.5 Ethics statement

This study was approved by the Ethics Review Committee of the Zhejiang CDC and was conducted in accordance with the principles of the Declaration of Helsinki. Written informed consent was obtained from all participants prior to their inclusion in the study.

## 3 Result

### 3.1 Participants characteristics

During the study period, a total of 5,225 questionnaires were distributed to eligible ILI patients. After applying the inclusion and exclusion criteria, 3,796 valid questionnaires were included in the final analysis, yielding an effective response rate of 72.7%. The primary reasons for exclusion were not meeting the ILI case definition (e.g., duration of illness >7 days), residing outside the surveillance area, or declining to participate.

These 3,796 enrolled patients had a nearly equal sex distribution, including 1894 male cases (49.89%) and 1902 female cases (50.11%). The median age was 76 years (IQR: 72–82 years), distributed across age groups as follows: 405 (10.7%) aged 60–69 years, 2093 (55.1%) aged 70–79 years, and 1,298 (34.2%) aged ≥80 years. Underlying medical conditions were present in 2568 patients (67.7%).

### 3.2 Influenza vaccination

Among 3,796 ILI cases, 1,258 (33.14%) received influenza vaccination during the current season (Table 1). Vaccination rates for the 2021–2022, 2022–2023, and 2023–2024 seasons were 32.10, 31.27, and 34.87%, respectively. Males had a significantly higher vaccination rate (34.88%) than females (31.40%; *p* = 0.023). Age-specific vaccination coverage was 25.43% (103/405) in the 60–69 age group, 35.36% (740/2093) in the 70–79 group, and 31.97% (415/1298) in the ≥80 group. Regarding vaccination history, 20.60% (782/3796) were vaccinated in both the current and previous seasons, 12.54% (476/3796) only in the current season, 9.72% (369/3796) only in the previous season, and 57.09% (2,167/3796) were unvaccinated in both seasons.

**Table 1:** ILI case vaccination characteristics and infection characteristics.

Characteristic	Total	Vaccination status		Statistics		Influenza detection		Statistics	
		Vaccinated	Unvaccinated	χ^2^	*p*	Influenza negative	Influenza positive	*χ* ^2^	*p*
No. (%)	3,796 (100)	1,258 (33.14)	2,538 (66.86)			3,152 (83.03)	644 (16.97)		
Season				χ^2^ = 4.67	0.097			χ^2^ = 159.70	< 0.001
2021–2022	835 (21.99)	268 (32.10)	567 (67.90)			809 (96.89)	26 (3.11)		
2022–2023	1,180 (31.09)	369 (31.27)	811 (68.73)			971 (82.29)	209 (17.71)		
2023–2024	1781 (46.92)	621 (34.87)	1,160 (65.13)			1,372 (77.04)	409 (22.96)		
Sex				χ^2^ = 5.18	0.023			χ^2^ = 0.00	0.959
Male	1894 (49.89)	661 (34.88)	1,234 (65.12)			1,572 (83.00)	322 (17.00)		
Female	1902 (50.11)	597 (31.40)	1,304 (68.60)			1,580 (83.07)	322 (16.93)		
Age group				χ^2^ = 16.30	< 0.001			χ^2^ = 30.84	< 0.001
60–69 y	405 (10.67)	103 (25.43)	302 (74.57)			305 (75.31)	100 (24.69)		
70–79 y	2093 (55.14)	740 (35.36)	1,353 (64.64)			1721 (82.23)	372 (17.88)		
≥ 80 y	1,298 (34.19)	415 (31.97)	883 (68.03)			1,126 (86.75)	172 (13.25)		
Underlying diseases^a^				χ^2^ = 0.66	0.416			χ^2^ = 0.34	0.558
Yes	2,568 (67.65)	840 (32.71)	1728 (67.29)			2,126 (82.79)	442 (17.21)		
No	1,228 (32.35)	418 (34.04)	810 (65.96)			1,026 (83.55)	202 (16.45)		
Vaccinated in the previous season				χ^2^ = 903.21	< 0.001			χ^2^ = 24.85	< 0.001
Yes	1,151 (30.32)	782 (67.94)	369 (32.06)			1,009 (87.62)	142 (12.38)		
No	2,645 (69.68)	475 (17.98)	2,167 (82.02)			2,142 (81.00)	503 (19.00)		
Vaccinated in the current season								χ^2^ = 36.12	< 0.001
Yes	1,258 (33.14)	-	-			1,110 (88.24)	148 (11.76)		
No	2,538 (66.86)	-	-			2042 (80.46)	496 (19.54)		

a Underlying diseases included a range of chronic conditions as reported by patients or recorded in medical charts, including but not limited to: asthma, chronic obstructive pulmonary disease, hypertension, coronary heart disease, diabetes, chronic kidney disease, cerebrovascular disease, and malignancy.

### 3.3 Influenza positivity

Among 3,796 ILI cases, 644 tested positive for influenza, with a positivity rate of 16.97%. Of these, 552 were influenza A infections, predominantly H3N2 (355 cases, 55.12% of positives), followed by H1N1 (188 cases, 29.19%). Influenza B was detected in 90 cases, mostly of the B/Victoria lineage (81 cases, 12.28%); the remaining positive cases were not subtyped. No significant difference in influenza positivity rates by sex was observed: 17.00% in males (322/1894) and 16.93% in females (322/1902) (*p* = 0.959). However, significant differences were observed across age groups. The positivity rate was highest among those aged 60–69 years (24.69%, 200/405), followed by 70–79 years (17.77%, 372/2093), and lowest in those aged ≥80 years (13.25%, 172/1298) (*p* < 0.001). The positivity rate was 17.21% among individuals with underlying diseases and 16.45% among those without, a difference that was not statistically significant (*p* = 0.558), as shown in Table 1.

### 3.4 Multivariate analysis

After adjustment for potential confounders, the analysis indicated that gender and age were significantly associated with influenza vaccination rates (*p* < 0.05). Specifically, women were significantly more likely to be vaccinated than men (aOR = 1.18, 95% CI: 1.03–1.35). Compared with adults aged 60–69, those in the 70–79 and ≥80 age groups had 1.62 (95% CI: 1.28–2.07) and 1.40 (95% CI: 1.09–1.80) times higher odds of being vaccinated, respectively (Table 2).

**Table 2:** Multivariate logistic regression analysis of influenza vaccination status.

Characteristic	OR	95% CI	*p*
Sex (Reference: Male)			
Female	1.18	1.03–1.35	0.020
Age group (Reference: 60–69 y)			< 0.001
70–79 y	1.62	1.28–2.07	
≥ 80 y	1.40	1.09–1.80	
Underlying disease (Reference: No)			
Yes	0.93	0.80–1.07	0.313

Using influenza virus positivity as the dependent variable, and age group, presence of underlying diseases, current-season vaccination status, and previous-season vaccination status as independent variables, a multivariable logistic regression analysis was performed. The results indicated that age, current-season vaccination, and previous-season vaccination were significantly associated with influenza positivity among ILI cases (*p* < 0.001). Age ≥ 70 years was identified as a protective factor. Compared with the 60–69 age group, the odds of influenza positivity were 0.70 times (95% CI: 0.54–0.90) in the 70–79 age group and 0.48 times (95% CI: 0.36–0.63) in the ≥80 age group. Compared to unvaccinated individuals, getting vaccinated against seasonal influenza can effectively reduce the risk of infection (OR = 0.63, 95% CI: 0.50–0.78). Similarly, previous-season vaccination also showed a protective effect (OR = 0.76, 95% CI: 0.61–0.96; *p* = 0.019), as shown in Table 3.

**Table 3:** Multivariate logistic regression analysis of influenza-positive ILI cases.

Characteristic	OR	95% CI	*p*
Sex (Reference: Male)			
Female	1.03	0.86–1.22	0.771
Age group (Reference: 60–69 y)			< 0.001
70–79 y	0.70	0.54–0.90	
≥ 80 y	0.48	0.36–0.63	
Underlying disease (Reference: No)			
Yes	1.11	0.92–1.33	0.284
Vaccinated in the current season (Reference: No)			
Yes	0.63	0.50–0.78	< 0.001
Vaccinated in the previous season (Reference: No)			
Yes	0.76	0.61–0.96	0.019

### 3.5 Vaccine effectiveness

**Figure 1:**
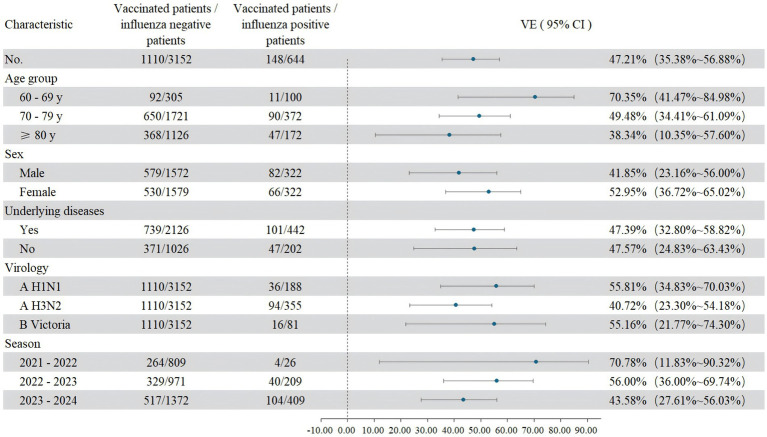
Influenza vaccine effectiveness (VE) in adults ≥60 years by demographic and clinical characteristics, Zhejiang Province, China, 2021–2024.

After adjusting for confounding factors such as gender, age group, underlying diseases, and flu season, the overall VE was 47.21% (95% CI: 35.38–56.88%). Subtype-specific VE was 55.81% (95% CI: 34.83–70.03%) against A/H1N1, 40.72% (95% CI: 23.30–54.18%) against A/H3N2, and 55.16% (95% CI: 21.77–74.30%) against B/Victoria. VE varied by age group: 70.35% (95% CI: 41.47–84.98%) in the 60–69 age group, 49.48% (95% CI: 34.41–61.09%) in the 70–79 group, and 38.34% (95% CI: 10.35–57.60%) among those aged 80 years or older. Across influenza seasons, VE was 70.78% (95% CI: 11.83–90.32%) in 2021–2022, 56.00% (95% CI: 36.00–69.74%) in 2022–2023, and 43.58% (95% CI: 27.61–56.03%) in 2023–2024, as shown in Figure 1.

**Figure 2:**
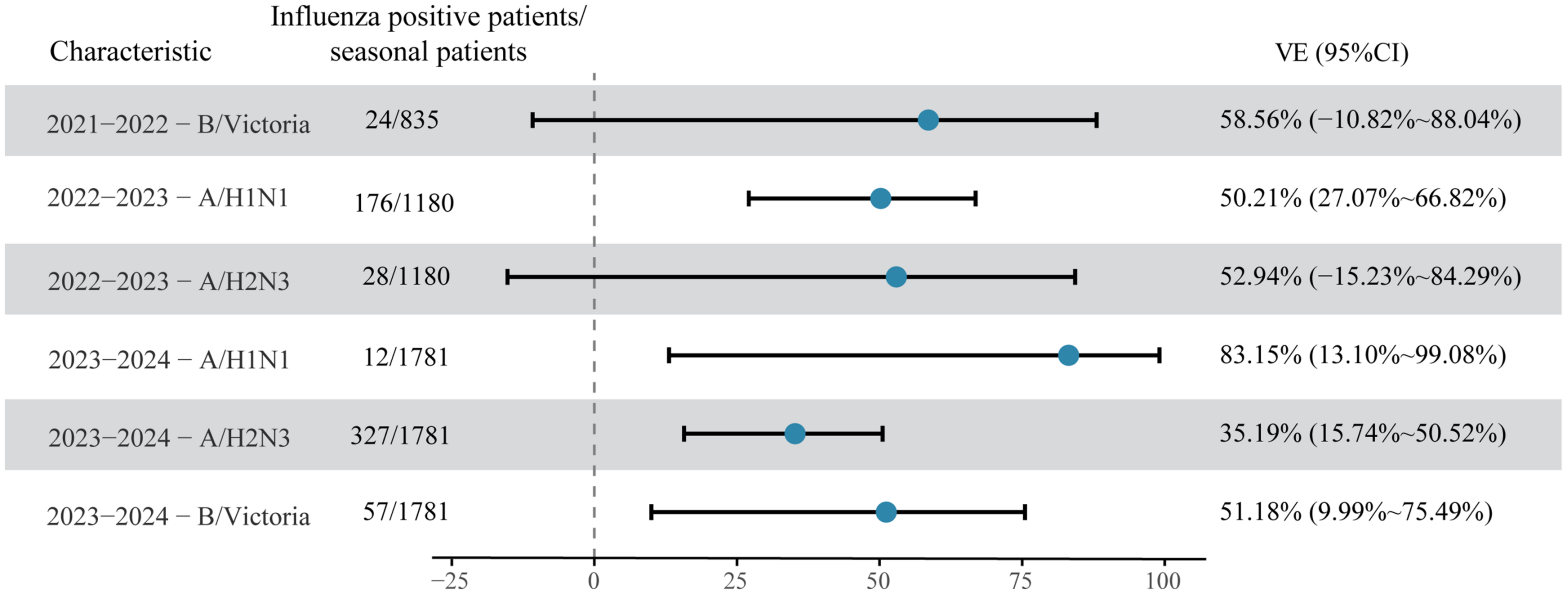
Stratified influenza vaccine effectiveness (VE) by season and predominant subtype among adults aged ≥60 years in Zhejiang Province, China, 2021–2024.

Further stratification by influenza subtype revealed additional heterogeneity. In the 2021–2022 season, all influenza cases were due to B/Victoria, with a VE of 58.56% (95% CI: −10.82- 88.04%). In 2022–2023, when both influenza A subtypes co-circulated, VE against A/H1N1 was 50.21% (95% CI: 27.07–66.82%) and against A/H3N2 was 52.94% (95% CI: −15.23-84.29%). In 2023–2024, VE was highest against A/H1N1 at 83.15% (95% CI: 13.1–99.08%), followed by B/Victoria at 51.18% (95% CI: 9.99–75.49%), and was lowest against the predominant A/H3N2 at 35.19% (95% CI: 15.74–50.52%), as shown in Figure 2. It should be noted that some estimates, particularly those for subtypes with a small number of positive cases (e.g., A/H1N1 in 2023–2024, *n* = 12), are associated with wide confidence intervals and should be interpreted with caution.

## 4 Discussion

This study assessed influenza VE in adults ≥60 years in Zhejiang over three seasons (2021–2024). The overall VE was 47.21%, with substantial variation by season (70.78, 56.00, 43.58%), subtype (higher for A/H1N1 and B/Victoria than A/H3N2), and age (declining from 70.35% in 60–69 years to 38.34% in ≥80 years). These findings demonstrate that while influenza vaccination provided moderate overall protection in this older population, its effectiveness was influenced by season, viral subtype, and markedly by age.

These findings hold particular significance given China’s rapidly aging population. By the end of 2023, individuals aged 60 and above accounted for 21.1% of the total population (12). Older adults face elevated risks of infection and severe outcomes from influenza. Vaccination remains a cornerstone of preventive strategy for this growing demographic, underscoring the need to understand its real-world effectiveness.

In China, influenza vaccination is included in the non-immunization program, and coverage remains low among older adults nationwide. During the 2021–2022 and 2022–2023 influenza seasons, the estimated influenza vaccination rates among adults aged ≥60 years were only 3.75 and 4.16%, respectively (13). Certain regions report even lower rates; for example, the highest annual vaccination rate in Henan Province between 2020 and 2022 was merely 3.15% (14), and in Anhui Province from 2021 to 2023, it was only 1.84% (15). In contrast, the present study found that the influenza vaccination rates among ILI patients aged ≥60 years were 32.10, 31.27, and 34.87% during the 2021–2022, 2022–2023, and 2023–2024 influenza seasons, respectively. These findings are consistent with a previous report showing an influenza vaccination rate of 36.95% among residents aged ≥50 years in Zhejiang Province in 2022 (16).

A study involving older adults aged 60 and above in Ningbo, Zhejiang Province from 2018–2019 to 2021–2022 found that the overall VE of influenza vaccines was 63.5% (95% CI: 56.3–69.5%) (17). A study conducted during the 2019–2020 influenza season showed that the VE of influenza vaccines among older adults aged 60 and above was 37.04% (95% CI: 9.43–56.23%) (18), and the VE found in our study was slightly higher. In this study, the overall VE of influenza vaccines in the three influenza seasons from 2021 to 2024 was 47.21%, and the VEs in the three seasons were 70.78, 56.00, and 43.58%, respectively. The notably high pooled VE in the 2021–2022 season (70.78%) coincided with a season of very low influenza activity, resulting in only 26 positive cases, all of which were influenza B/Victoria. Consequently, this estimate is highly unstable, as reflected by its wide confidence interval. In the 2022–2023 season, with increased activity yielding 209 positive cases. However, subtype-specific estimates for A/H1N1 (*n* = 176) and A/H3N2 (*n* = 28) in this season remain based on limited numbers and should be interpreted with caution. The 2023–2024 season provided the largest sample (409 positive cases) and the most stable estimates. The overall VE was 43.58%, with the lowest point estimate observed against the predominant A/H3N2 subtype (35.19%). In contrast, the VE against A/H1N1 was 83.15%, but this is based on only 12 positive cases and has an extremely wide confidence interval, precluding any reliable conclusion about high effectiveness against this subtype. These findings, particularly the moderate overall protection and lower effectiveness against A/H3N2, are consistent with a recent large-scale, population-based TND study conducted in Southern China during the same season (19). That study, which included over 200,000 participants, reported an overall VE of 49.4% (95% CI: 47.8–50.9%) against laboratory-confirmed influenza. It also found lower VE against influenza A (41.9%) compared to influenza B (59.9%), aligning with our observation of lower effectiveness against the predominant A/H3N2 subtype.

Our pooled VE against influenza A (H1N1: 55.81%; H3N2: 40.72%) is broadly comparable to the VE of 51% against any influenza A reported in Italian older adults (≥65 years) during the 2023–2024 season (20). However, our estimates are substantially higher than those reported from other settings. For the 2022–2023 season, our adjusted overall VE was 56.00%, with subtype-specific estimates of 50.21% against A/H1N1 and 52.94% against A/H3N2. These figures are substantially higher than the VE of 29% (95% CI: 12–42%) against any influenza A reported in a European multi-country study among individuals aged ≥65 years during the same season (21). Similarly, a US study focusing on influenza-associated hospitalization in 2022–2023 reported a VE of 28% in adults aged ≥65 years (22). For the 2023–2024 season, our overall VE was 43.58%, with the lowest point estimate observed against the predominant A/H3N2 subtype (35.19%). However, early estimates from South Korea for 2023–2024 indicated a non-significant VE of 13.5% (95% CI: −17.9 to 36.6%) in adults aged ≥65 years (23).

The relatively higher VE observed in our study may have been influenced by a better antigenic match between the vaccine strains and the locally circulating influenza viruses during the study period. Although detailed local genetic characterization data are limited, national surveillance reports suggested a generally good match for the predominant lineages in these seasons. A meta-analysis showed that the VE in the case of matching between the prevalent strain and the vaccine strain (44.38, 95% CI: 22.63–60.01%) was higher than that in the case of mismatch (20.00, 95% CI: 3.46–33.68%) (24). Additionally, the distinct history of prior influenza exposures and vaccinations in our population could have shaped a different baseline immunity, potentially modulating the response to the current season’s vaccine (25).

We observed a marked decline in VE with increasing age. The VE of the 60–69 age group was 70.35%, that of the 70–79 age group was 49.48%, and that of the ≥ 80 age was 38.34%, showing a trend that the protective effect of the vaccine decreases with age. This pattern may be attributed to immunosenescence; namely, the age-related deterioration of immune function commonly observed in older populations (26).

The impact of repeated influenza vaccination on VE remains a subject of ongoing debate. This study found no significant difference in protection between those vaccinated only in the current season and those vaccinated repeatedly. However, compared with individuals unvaccinated in both seasons, both single-season and repeated vaccination provided measurable protection to older adults, which is consistent with previous meta-analyses (27).

### 4.1 Limitations

The findings of this study are primarily derived from data collected from influenza sentinel hospitals in Ningbo and Jiaxing cities of Zhejiang Province. Therefore, the research results have certain limitations when applied to different regions and populations across the province and even the country. Furthermore, as the data were obtained from sentinel surveillance systems, selection bias may exist due to the underrepresentation of individuals with mild or asymptomatic infections who did not seek medical care. Notably, some season-subtype strata contained very few positive cases. VE estimates derived from such small samples are inherently unstable and have wide confidence intervals, which may lead to overestimation or imprecise representation of the true protective effect. Additionally, a precise assessment of the antigenic match between the vaccine strains and the locally circulating influenza viruses for each season could not be conducted due to the unavailability of detailed local viral genetic characterization data.

## 5 Conclusion

Influenza vaccination provides a moderate overall protective effect against influenza infection in adults aged 60 years and above. Both age and vaccination status were identified as significant factors influencing influenza infection. VE varied across influenza subtypes, age groups, and epidemic seasons, and showed a declining trend with increasing age, particularly among individuals aged 80 years or older, where protection was more limited. To further optimize immunization strategies for older adults, future large-scale, multi-center, and long-term real-world studies are recommended to develop more targeted and differentiated vaccination policies.

## Data Availability

The original contributions presented in the study are included in the article/supplementary material, further inquiries can be directed to the corresponding authors.
